# A Web-Based, Population-Based Cirrhosis Identification and Management System for Improving Cirrhosis Care: Qualitative Formative Evaluation

**DOI:** 10.2196/27748

**Published:** 2021-11-09

**Authors:** Sarah J Javier, Justina Wu, Donna L Smith, Fasiha Kanwal, Lindsey A Martin, Jack Clark, Amanda M Midboe

**Affiliations:** 1 Center for Innovation to Implementation (Ci2i) VA Palo Alto Health Care System Menlo Park, CA United States; 2 Center for Innovations in Quality, Effectiveness and Safety (IQuESt) Michael E. DeBakey VA Medical Center Houston, TX United States; 3 Section of Gastroenterology and Hepatology Baylor College of Medicine Houston, TX United States; 4 Section of Health Services Research Baylor College of Medicine Houston, TX United States; 5 National Institute of Environmental Health Sciences (NIEHS) Division of Extramural Research & Training Population Health Branch Research Triangle Park, NC United States; 6 Department of Health Law, Policy, and Management School of Public Health Boston University Boston, MA United States; 7 Stanford School of Medicine Stanford, CA United States

**Keywords:** cirrhosis, informatics, care coordination, implementation, Consolidated Framework for Implementation Research (CFIR), quality improvement

## Abstract

**Background:**

Cirrhosis, or scarring of the liver, is a debilitating condition that affects millions of US adults. Early identification, linkage to care, and retention of care are critical for preventing severe complications and death from cirrhosis.

**Objective:**

The purpose of this study is to conduct a preimplementation formative evaluation to identify factors that could impact implementation of the Population-Based Cirrhosis Identification and Management System (P-CIMS) in clinics serving patients with cirrhosis. P-CIMS is a web-based informatics tool designed to facilitate patient outreach and cirrhosis care management.

**Methods:**

Semistructured interviews were conducted between January and May 2016 with frontline providers in liver disease and primary care clinics at 3 Veterans Health Administration medical centers. A total of 10 providers were interviewed, including 8 physicians and midlevel providers from liver-related specialty clinics and 2 primary care providers who managed patients with cirrhosis. The Consolidated Framework for Implementation Research guided the development of the interview guides. Inductive consensus coding and content analysis were used to analyze transcribed interviews and abstracted coded passages, elucidated themes, and insights.

**Results:**

The following themes and subthemes emerged from the analyses: outer setting: needs and resources for patients with cirrhosis; inner setting: readiness for implementation (subthemes: lack of resources, lack of leadership support), and implementation climate (subtheme: competing priorities); characteristics of individuals: role within clinic; knowledge and beliefs about P-CIMS (subtheme: perceived and realized benefits; useful features; suggestions for improvement); and perceptions of current practices in managing cirrhosis cases (subthemes: preimplementation process for identifying and linking patients to cirrhosis care; structural and social barriers to follow-up). Overall, P-CIMS was viewed as a powerful tool for improving linkage and retention, but its integration in the clinical workflow required leadership support, time, and staffing. Providers also cited the need for more intuitive interface elements to enhance usability.

**Conclusions:**

P-CIMS shows promise as a powerful tool for identifying, linking, and retaining care in patients living with cirrhosis. The current evaluation identified several improvements and advantages of P-CIMS over current care processes and provides lessons for others implementing similar population-based identification and management tools in populations with chronic disease.

## Introduction

### Background

Over 4.5 million US adults were diagnosed with liver disease in 2018, and an estimated 44,358 died of chronic liver disease in 2019 [1,2]. Recent estimates indicate that the total annual health care cost of advanced liver disease or cirrhosis is close to US $9.5 billion [3]. Cirrhosis may be accompanied by a host of complications that require a complex, patient-centered treatment plan, including biannual surveillance visits [4,5]. However, patients with cirrhosis are often not diagnosed by their primary care provider or, alternatively, are not referred to specialty care after diagnosis [6]. Owing to the complexity of cirrhosis treatment, a systematic approach to cirrhosis management is critical to preventing further complications and the risk of death.

Health informatics tools have shown promise in the management of complex diseases and can enhance the continuity of care [7-10]. However, it is often challenging to incorporate these tools into existing clinical workflows, with implementation success dependent on ongoing usability assessments [11,12]. In view of the high morbidity and mortality associated with cirrhosis, we conducted a preimplementation formative evaluation to identify facilitators and barriers to implementation of the Population-Based Cirrhosis Identification and Management System (P-CIMS) [13].

### P-CIMS

**Figure 1:**
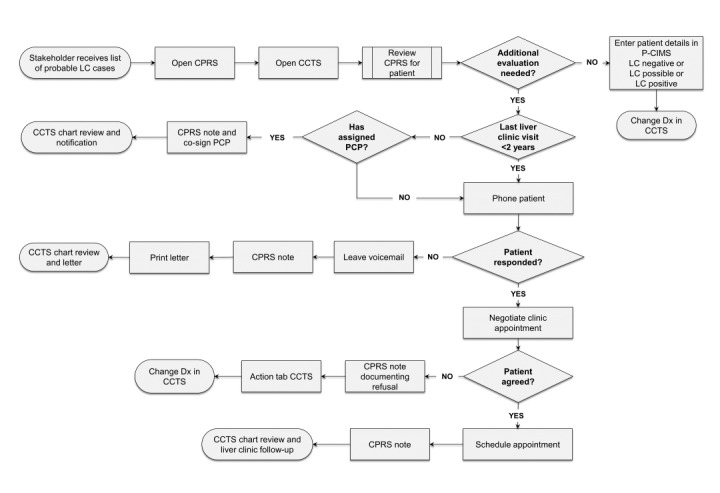
P-CIMS process workflow map (adapted from Kanwal et al [13]). CPRS: Computerized Patient Record System; CCTS: cancer case tracking system; Dx: diagnosis; LC: liver cirrhosis; P-CIMS: Population-Based Cirrhosis Identification and Management System; PCP: primary care provider.

P-CIMS, a secure, web-based informatics tool for providers, is designed to ensure efficient cirrhosis case identification in clinical populations, facilitate coordination of care for known cases, and prevent loss to follow-up [13]. Figure 1 presents the overall process workflow chart for P-CIMS. In Figure 1, we describe a scenario in which a provider receives a list of probable cirrhosis cases and must confirm cirrhosis diagnosis before tracking them in P-CIMS.

#### Development and Testing

The precursor to P-CIMS, the Cancer Care Tracking System (CCTS), was developed by an interdisciplinary team of clinicians, programmers, and informatics experts in 2007 [9]. CCTS was a tool that consolidated local Veterans Health Administration (VHA) electronic medical record (EMR) data into a dashboard on which providers could view abnormal lung or liver cancer images and then track the clinical steps required to reach a definitive diagnosis and treatment plan.

In 2015, P-CIMS was developed using CCTS as a base platform and then improved over 9 months [13]. P-CIMS data elements were validated using the full EMR from the VHA National Corporate Data Warehouse as the reference standard. The beta version described by Kanwal et al [13] was tested between 2015 and 2017 at a site with over 3800 patients diagnosed with cirrhosis, according to the EMR data. Results from the beta test indicated that the use of P-CIMS resulted in 30% of probable cases being referred to liver specialty care. Findings from the beta version testing version of P-CIMS have been published elsewhere [13].

#### Functional Components

There are 2 main functional components of P-CIMS:

1. Generation of lists of patients who are likely to have undiagnosed cirrhosis and patients with diagnosed cirrhosis who have been lost to follow-up using EMRs; and

2. Task management features designed to facilitate retention in care and help monitor whether patients complete recommended surveillance testing.

##### Generation of Lists of Probable Cirrhosis Cases

The P-CIMS report generator allows providers to generate lists of patients who are either likely to have undiagnosed cirrhosis or who have diagnosed cirrhosis but who have been lost to follow-up (henceforth, referred to as *probable cases*). Providers can customize these lists based on the following criteria:

1. Any outpatient or inpatient encounters in the last 3 years in which the patient had at least one cirrhosis diagnosis, as designated by the validated *International Classification of Diseases, Ninth Revision* and *Tenth Revision* codes [14];

2. Possible cirrhosis, defined as either aspartate aminotransferase to platelet ratio index >2.0, or Fibrosis-4 index >3.24 in patients with an active hepatitis C virus (HCV) infection.

3. Last visit to a liver or HCV clinic is >180 days earlier (ie, more than 6 months ago)

Probable cases must be confirmed by their provider via chart review for each patient on the list to confirm whether the patient needs to be coded in P-CIMS for cirrhosis surveillance tracking (Figure 1).

##### Cirrhosis Tracking and Management

**Figure 2:**
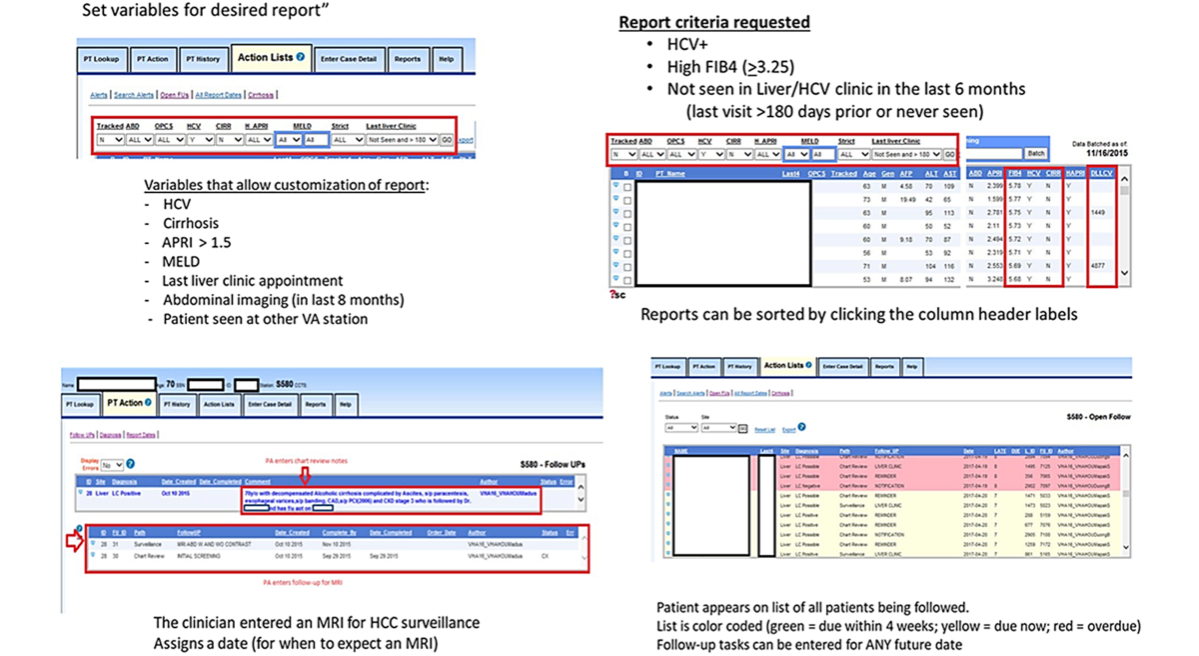
Static screenshots from the Population-Based Cirrhosis Identification and Management System, adapted from Kanwal et al [13] (top 2 images: generation of probable cases list; bottom 2 images: cirrhosis tracking). APRI: aminotransferase to platelet ratio index; FIB4: fibrosis-4; HCC: hepatocellular carcinoma; HCV: hepatitis C virus; MELD: model for end-stage liver disease; MRI: magnetic resonance imaging; VA: Department of Veterans Affairs.

The second function of P-CIMS is to track and manage all aspects of ongoing cirrhosis care for probable cases confirmed via chart review. Providers can track patient compliance with recommended consultations, screenings, surveillance testing, imaging, follow-up visits, or referral for transplant evaluation (Figure 2 shows static screenshots from P-CIMS).

### Purpose of This Study

The purpose of this study is to conduct a preimplementation formative evaluation using qualitative interviews to identify barriers and facilitators of P-CIMS implementation across 3 testing sites, including at the beta testing site.

## Methods

### Setting

We conducted a qualitative formative evaluation with key stakeholders at 3 high-volume Department of Veterans Affairs (VA) medical centers in the Sierra Pacific and Southern Central United States (N=10), including a high-volume site at which beta testing occurred. The other 2 sites were chosen based on the criteria for successful P-CIMS implementation. These criteria included the following: (1) established relationships between external facilitators (FK, DLS, and AMM) and key stakeholders; (2) internal facilitators, such as primary care and liver specialty clinical leadership support; and (3) liver clinic patient volume. The sites that were chosen were known to have a higher than average liver clinic volume.

### Sample

Although the key purpose of this study was to identify potential implementation barriers and facilitators through qualitative interviews, we were also interested in examining usability. As such, we centered on interviews with specific end-users who would be using the tool for clinical care; we followed sample size recommendations from the empirical literature. Guidance by Turner, Lewis, and Nielsen maintains that a minimum of 3 to 5 participants are sufficient for a usability test with the caveat that running additional subjects during the same test is unlikely to reveal new information [15]. Further, other guidance from the quality improvement literature maintains that code saturation can be reached with as few as 9 interviews [16]. Our final sample comprised 10 providers, including 8 physicians and midlevel providers from liver-related specialty clinics and 2 primary care providers who managed patients with cirrhosis.

### Design

#### Interview Guide Development and Testing

We created a semistructured interview guide based on the Consolidated Framework for Implementation Research (CFIR), a metatheoretical framework that describes a set of constructs organized within 5 domains [17]. These constructs allow implementation scientists to systematically assess barriers and facilitators to implementation, including contextual factors relevant to multisite implementation projects. The following CFIR domains were chosen for P-CIMS evaluation: outer, inner, and individual characteristics.

The interview guide was developed, iteratively modified, and pilot-tested by 2 qualitative researchers with training in medical anthropology and public health (LAM and JC). The pilot interview participants were gastroenterologists from the P-CIMS beta test site and a gastroenterologist from a nontest site who had previously seen a demo version of P-CIMS. Feedback from these interviewees was used to refine the interview guide.

#### Semistructured Interviews

The qualitative team conducted semistructured interviews between January and May of 2016. Providers were asked about current practices for identifying and linking patients with cirrhosis to liver clinic specialty care. Interview topics included the perceived value and benefit of P-CIMS, usability, barriers to implementation, and opportunities to improve the tool. VHA privacy rules prevented live demonstration of the 2 nontest sites. For those interviews, a Microsoft PowerPoint demonstration, including deidentified static screenshots of P-CIMS, was shown to the interviewees. An expert was available during the interviews to answer questions about the tool.

At the beta test site, the interviews included content-specific usability questions. Four providers who had already used the beta version of P-CIMS were asked questions that explored how they had integrated P-CIMS into their clinical workflow, challenges in using the system, and suggestions for improvement (LAM and JW). Participants were asked to open P-CIMS and walk through how they used the system while noting down any features that were helpful or not helpful.

### Analytic Approach

Content analysis is the primary approach used in the current evaluation [18]. Two analysts (LAM and JW) independently reviewed the transcripts and constructed the codes to describe the data. An iterative consensus process was used to draft a list of subcodes, exemplar quotes, and broader code categories. This list was then used to create a codebook consisting of a priori codes that emerged from the data (eg, *suggestions for improving P-CIMS*) and code definitions [18]. Individual codes were also deductively linked to CFIR constructs that guided the data collection. For example, *suggestions for improving P-CIMS* was linked to the CFIR construct *Inner Setting: Readiness for Implementation*. These constructs served as sensitizing concepts to guide coding, but still allowed for the identification of other salient themes in the data [18].

The codebook was applied for consensus-based coding of the remaining transcripts. Two coders (LAM and JW) met regularly to identify additional codes and refine existing code definitions, with any discrepancies resolved through negotiated consensus. One member of the qualitative team (JW) compiled coded passages into separate transcripts, which were used to derive subthemes. Subthemes were then compiled into matrices for each code, which included exemplar quotes and examples. For example, for the major code *suggestions for improving P-CIMS*, one subtheme was *adding patients to P-CIMS*, with descriptive examples, *automatic instead of manual entry*, and *need clear definition of cirrhosis*.

Several steps were taken to bolster the validity of data collection and analysis. First, 2 pilot interviews with the target users of P-CIMS were used to refine the initial codebook (See *Interview Guide Development and Testing* section). This is a step that is often skipped in qualitative research, but it allows investigators to address instrumentation and bias issues before actual data collection occurs [19]. Second, the qualitative team purposively sampled interview participants and interviewed only individuals who would provide the most appropriate and meaningful insights on P-CIMS [20]. To that end, 20% (2/10) primary care providers were interviewed, and specialists (eg, gastroenterologists) comprised most of the sample (8/10, 80% providers). These specialists are the target end-users for P-CIMS, as they work most closely with patients who would benefit from the tool. In contrast, we found that interviews with the 2 primary care providers did not offer unique information in comparison to specialty care providers. Therefore, we chose not to interview primary care providers. Third, the qualitative team employed 2 types of triangulation of analytic findings: theoretical triangulation and investigator triangulation [21]. Theoretical triangulation, defined as the use of substantive theoretical lenses (ie, CFIR) to drive data collection and review research findings, provided a strong basis for the development of both the interview guide and the codebook. Investigator triangulation, or data analysis by more than 2 independent coders with experience in qualitative research (ie, LAM, JW, and DLS) ensured that concurrence for the final list of subthemes and descriptive examples was achieved. Data analyses were iterative and continued until thematic saturation was reached.

### Ethics

The Stanford University Human Research Protection Program reviewed and approved this project as a quality improvement project. All participants provided verbal consent before being interviewed. For this type of study (ie, quality improvement), formal consent is not required.

### Human Rights

All procedures performed in studies involving human participants were in accordance with the ethical standards of the institutional and national research committee and with the 1964 Helsinki declaration and its later amendments or comparable ethical standards. The Stanford University Human Research Protection Program reviewed and approved this project as a quality improvement project. The VA Central Institutional Review Board also approved project #14-11 Population-based identification and management of veterans with HCV cirrhosis. For this type of study (ie, quality improvement), formal consent was not required.

### Informed Consent

All participants gave verbal consent before being interviewed.

## Results

### Sample

Nine interviews took place in person or over the phone and were audio-recorded and transcribed. One interviewee requested that the interview not be recorded, and extensive notes were taken for this interview.

### CFIR Mapping

**Figure 3:**
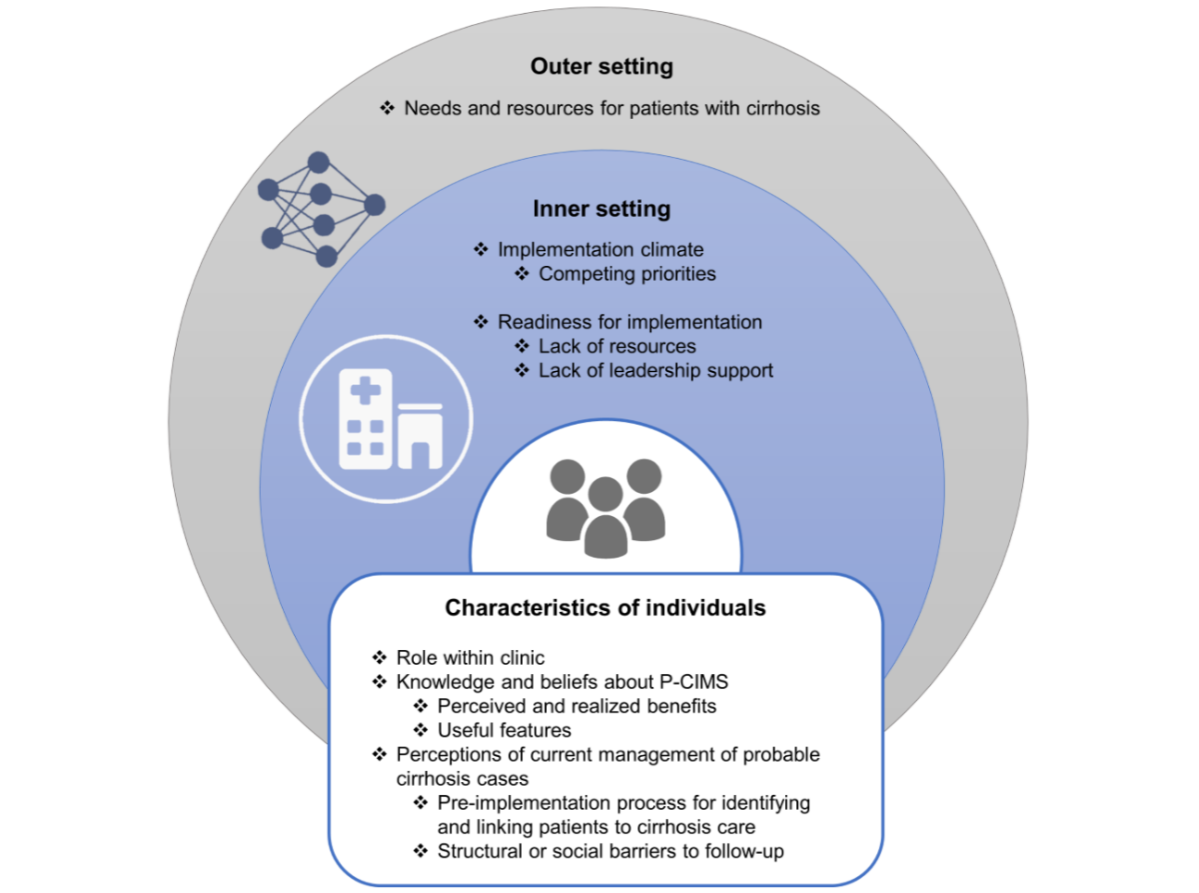
Results of qualitative analysis mapped to Consolidated Framework for Implementation Research constructs. P-CIMS: Population-Based Cirrhosis Identification and Management System.

Table 1 presents the main findings mapped to the CFIR constructs of the outer and inner settings and individual characteristics of the individuals. Figure 3 presents the main findings mapped to these CFIR constructs in a descriptive figure.

**Table 1:** Consolidated framework for implementation research constructs in Population-Based Cirrhosis Identification and Management System implementation.

CFIR^a^ construct	Example from evaluation	Illustrative quote
Outer setting domain—needs and resources for patients	Health informatics tools like P-CIMS^b^ can enhance care continuity for patients with cirrhosis	“If patients are lost to follow-up for 2 years, they drop off our panel…we don’t currently have any way of…reaching out to them like the cirrhosis tracker would do.”
Inner setting domain—readiness for implementation	Providers said that lack of resources might prevent them from integrating P-CIMS in their clinical workflow	“We don’t necessarily have the time in our day-to-day duties to really do justice to what this tool really can do.”
Inner setting domain—implementation climate	Leadership is not as engaged with cirrhosis initiatives as other initiatives at their location	“…we have problems getting enough support for HCV^c^ care…cirrhosis is not on the radar.”
Characteristics of individuals —role within clinic	Providers perceive that cirrhosis tracker implementation is not a part of their role	“If we had a very clear algorithm that the liver clinic could sign off on, then potentially the nurses could work through that but you’d have to work with the nursing service to their agreement that it was within their scope.”
Characteristics of individuals —knowledge and beliefs about the intervention	P-CIMS can be used to streamline continuity of care for patients with cirrhosis	“…it could very well change the landscape of hepatology, cirrhosis care as we know it. Anything that we can do that’s going to be innovative and…improve access and quality—typically is adopted.”
Characteristics of individuals —perceptions of current practices	Manual tracking systems for patients with cirrhosis are ineffective	“We used to do liver lesion monitoring where we manually entered patients into Excel on a monthly basis. This didn’t work well…we no longer do this, use the dashboard instead.”

^a^CFIR: Consolidated Framework for Implementation Research.

^b^P-CIMS: Population-Based Cirrhosis Identification and Management System.

^c^HCV: hepatitis C virus.

#### Characteristics of Individuals: Role Within Clinic

Overall, 50% (5/10) of the interviewees discussed how conflicting role expectations may hinder P-CIMS implementation. For example, liver clinic specialists expressed concern that primary care providers may not be as well versed in cirrhosis management as liver or infectious disease specialists. One provider said:

[The tool] makes sure that the referring providers are aware that these patients have risk factors for advanced fibrosis cirrhosis and underlying Hepatitis C and need to be seen in our service.

ID6

Furthermore, some providers said that without an automated reminder system, scheduling staff may forget to contact patients for follow-up appointments. One provider said:

The liver clinic relies on automated CPRS callback and reschedule procedures. Patients who are due to return to clinic receive an automated phone reminder and an auto-generated appointment reminder card prior to the expected clinic visit date. Similar procedures are in place for when a patient is a “no-show.” No one is assigned to track follow-up beyond the automated process.

ID8

#### Characteristics of Individuals: Perceptions of Current Practices

##### Preimplementation Process for Identifying and Linking Patients to Cirrhosis Care

Overall, 60% (6/10) of the providers discussed the current processes for identifying and linking patients to cirrhosis care. Before P-CIMS implementation, liver clinic staff relied on referrals from primary care or infectious disease clinics for the identification of patients with cirrhosis. After a patient with cirrhosis was linked to the liver specialty clinic, some liver clinic providers created manual tracking systems to manage patient follow-up office visits and surveillance testing. However, as one provider explained, these tools could be cumbersome for the staff to maintain:

So the group of patients we used to track in the past are people who had abdominal imaging results...we actually have a list that we follow to make sure that they get the proper form of imaging...enter them, manually enter them into spreadsheets and then we actually make sure that every 6 months they have the imaging....But we had to stop doing that because we just don’t have any manpower to be able to do that.

ID4

##### Structural and Social Barriers to Follow-Up

Overall, 70% (7/10) of the providers cited various reasons for patients with cirrhosis not attending follow-up appointments or not being linked to care after their initial appointment. Some barriers are structural or social in nature. For example, even though the VA routinely sent automated telephone appointment reminders to patients, many did not keep their appointments. Providers cited lack of response as attributable to potential barriers such as homelessness, drug addiction, or lack of access to mail or a phone. Second, if a patient canceled an appointment, there was no alert system in the EMR to notify the provider or to remind the administrative staff that the patient needed rescheduling. A third reason for loss to follow-up, as reported by providers, was the characteristics of the patients themselves. For instance, patients in the early stages of cirrhosis may be asymptomatic. These patients may not monitor their condition closely and, consequently, may stop attending ultrasound and other appointments. For instance, one provider stated:

Everything can be perfect at our end, but he or she would not show up for [an appointment]. So education is the first and foremost thing for them to understand the gravity of the condition and the importance of follow-up even though they feel well.

ID1

In addition, many patients with multiple comorbidities may not prioritize cirrhosis when other health issues cause immediate discomfort. Finally, providers reported that some patients may choose not to pursue any treatment for their liver conditions.

#### Inner Setting Barrier: Readiness for Implementation

Overall, 50% (5/10) of the interviews discussed clinical readiness for implementing P-CIMS. Overall, providers felt that P-CIMS would be best suited for use in a liver clinic or other specialties that regularly identified and managed patients with cirrhosis. Other providers cited barriers such as the lack of available time and number of staff needed to learn and use the tool, management of cirrhosis as not being part of primary care focus, and the worry that P-CIMS would add to the workload burden. For instance, one provider said:

I’m just a little bit nervous about any software that’s going to require a lot of extra action. [Primary care] is not going to review 1200 patients to see who is a candidate for this. Providers are pretty burnt out.

ID5

Participants acknowledged that it would take some time and training before some services fully embraced P-CIMS. Providers whose local decision-makers valued innovative tools and their potential to improve patient care felt that P-CIMS was likely to be adopted in their setting. However, the system would need complete buy-in from all providers as well as thoughtful planning about how to best integrate it into the current workflow.

##### Lack of Resources

Overall, 50% (5/10) of the providers pointed out the lack of adequate staff time and resources as the biggest obstacle to implementing and using P-CIMS. As one nurse practitioner noted:

We don't necessarily have the time in our day-to-day duties to really do justice to what this tool really can do. So finding the time, carving out the time, the uninterrupted time to be thorough in doing this is the issue.

ID6

Participants said that P-CIMS needed a designated coordinator to coordinate and send reminders to primary care for follow-up. However, most interviewees stated that such a coordinator would need additional support. One provider said:

Either we have one person who is 100% dedicated to putting in all of the information regarding all of the patients that are being seen in the tracker and then put their follow-up times, date, and whatever things we need...Even that person may or may not be able to do all of the things that are necessary because the workload is going to be significant.

ID10

In addition, the participants said that adding P-CIMS duties would slow down their productivity. Reports of chart review times, along with accompanying patient telephone contact activities, were closer to 12 to 13 minutes instead of 7 minutes, as originally estimated by the initial beta testers. Some providers also said that any new patients identified through P-CIMS would increase the demand for other hospital services such as radiology, procedures, and laboratory tests. To address this limitation, one option proposed by a provider was to distribute the P-CIMS duties among several staff members. They stated:

On our [Patient Aligned Care Team] team, we have an LVN and an RN and a pharmacist...I think all 3 of them, you know, have some time when I give them specific tasks, like dashboard tasks to do, I think they can do it.

ID12

One caveat to this strategy is that it involves a high level of coordination to ensure that inefficiencies and redundancies do not occur.

Ideas varied about the best way to integrate the use of P-CIMS into the clinic workflow. Most interviewees agreed that the user would need uninterrupted time to use the P-CIMS accurately. With the current state of resources, providers said that uninterrupted time would most likely be after hours or on weekends. As an alternative approach, one provider suggested entering patient data into the tool at the end of a visit, when the patient’s information is fresh in the provider’s mind. They stated:

I think it probably would require anywhere from 3 to 5 minutes to complete [patient tracker] because you are fresh and then you are actually looking at the patient record and you’ve just completed the notes.

ID10

Estimates varied across providers as to how often the tool would need to be beneficial.

Some interviewees said that the tool should be used once every 3 to 6 months, whereas others said that once per month use would be sufficient.

##### Lack of Leadership Support

A total of 40% (4/10) of the providers discussed the lack of leadership support for tools such as P-CIMS. One provider noted that cirrhosis was not a leadership priority at their location, and that buy-in for P-CIMS would require more leadership engagement. They said:

If it’s a financially productive activity... [administration] might be willing to provide support. You’re not gonna save money by getting more patients into hepatitis C treatment, so you know, it’s sort of a bottom line for the administration....The motivation would have to be better patient care. It couldn’t be a financial motivation.

ID12

To achieve this, the provider recommended highlighting the tool’s potential to improve patient care to site leadership.

#### Inner Setting Barrier: Implementation Climate

##### Competing Priorities

A total of 70% (7/10) providers discussed competing resources at their sites. The competing priorities were similar across the primary care providers at different sites. Providers stated that the treatment of chronic conditions, such as heart failure or diabetes, took precedence over cirrhosis. In addition, providers described having too many dashboard tools to use. For instance, one provider said:

We already have an opioid dashboard. We have an endocrine dashboard. We have diabetes, we have hypertension, and to be honest, almost nobody has time to actually look at them.

ID5

Most of these tools are not regularly used by providers because of time constraints, or because they find it confusing and onerous to use multiple tools. Finally, as mentioned in most interviews, current staffing levels and clinic duties made it difficult to find time to incorporate a new tool into the current workflow.

#### Characteristics of Individuals: Knowledge and Beliefs

##### Perceived and Realized Benefits

Most participants (ie, 8/10, 80%) generally understood and valued P-CIMS’s ability to link patients who are most likely to fall through the cracks or who are at the highest risk of cirrhosis to care. One provider said:

We [infectious disease clinic] are more focused on treating patients before they develop cirrhosis, so for me the focus will be Hepatitis C and Hepatitis B infected patients, identifying them before they have cirrhosis, and the tracker allows this to happen.

ID9

Other providers felt that the P-CIMS’s ability to identify these patients and help providers monitor and follow up with patients using reminders and task lists was its most valuable benefit. Some providers even saw the potential for P-CIMS to be used for other chronic conditions such as diabetes, high blood pressure, and heart failure. For instance, one provider said:

I think also it can be used to help in other disease processes, not just liver. Because it has the upside then of monitoring diabetes, monitoring your high blood pressure, possibly monitoring patients with COPD, CHF.

ID6

Many found it more powerful and user-friendly than existing tools such as the clinical case registry in its ability to store, track, and organize information about patients.

##### Useful Features

Most providers (ie, 7/10, 70%) stated that certain P-CIMS features were useful. Providers found the ability to set reminders and track patient follow-up actions to be one of the most useful aspects of P-CIMS. Functions such as sorting capabilities and actions color-coded by priority were also cited as helpful. In addition, providers also liked the following features: radiology reports, calculated measures of cirrhosis risk (eg, model for end-stage liver disease score, Fibrosis-4 index for liver fibrosis, aspartate aminotransferase to platelet ratio index), primary care physician reminders to place consults**,** cancer tracking options**,** patient look-up, action lists, and capturing barriers to care. Suggestions for P-CIMS improvement

##### Suggestions for Improvement

All interviewees (ie, 10/10, 100%) provided suggestions for improving P-CIMS. Providers’ suggestions for P-CIMS improvement were categorized into 4 themes: changes that would buffer against a slowdown of clinic productivity, changes to existing content, usability improvements, and potential new features. Table 2 presents these suggestions based on theme.

**Table 2:** Provider suggestions for Population-Based Cirrhosis Identification and Management System improvement by theme.

Avoiding productivity slowdown	Changes to existing content	Usability improvements	Potential new features
Use P-CIMS^a^ outside of clinic hours or on weekends Use P-CIMS after completing patient notes	Provide relevant information in prominent areas of P-CIMS Provide a clear definition of cirrhosis on P-CIMS to guide decision-making Provide a guide for midlevel providers diagnosis cirrhosis	Include patient information (eg, telephone number) in the tool to bypass referring to the EHR^b^ Autopopulate P-CIMS with EHR data Implement an autosave feature to avoid loss of work Incorporate tracking of clinical surveillance guideline requirements Track use metrics (eg, the amount of time each user spends on P-CIMS) Incorporate a filter feature where patients can be filtered by providers Autopopulate follow-up dates	Add search feature for abbreviations or acronyms Add at-a-glance tab Add a pending tab Add quick-access buttons Add an indicator for a closed consultation

^a^P-CIMS: Population-Based Cirrhosis Identification and Management System.

^b^EHR: electronic health record.

## Discussion

### Principal Findings

A preimplementation formative evaluation using the CFIR domains of outer setting, inner setting, and characteristics of individuals identified several key benefits and barriers to implementing a health informatics tool for the management of patients with cirrhosis—P-CIMS—at 3 sites. While participants expressed overall interest in and appreciation for the potential value of P-CIMS, there was concern about the feasibility of implementation. Mainly, barriers in the inner setting—understaffing and workload—posed the biggest challenges in the implementation of P-CIMS.

During the formative evaluation process, providers discussed current strategies for linking patients with cirrhosis to liver clinic specialty care (characteristics of individuals: perceptions of current management of patients with cirrhosis). In the absence of P-CIMS, providers used manual spreadsheets and manual tracking systems to manage scheduled office visits, surveillance testing, and care. For some clinics, the integration of digital tools in addition to regular electronic health record use may be onerous and result in less efficient forms of documentation. For instance, the use of electronic health records to conduct clerical and administrative tasks has been shown to comprise nearly half of some clinicians’ overall workdays [22]. Providers burdened with these tasks may seek out alternatives to P-CIMS to track patients with cirrhosis. Overreliance on nonelectronic health record documentation methods, such as noncoded text notes, has been shown in the past studies of health data [23]. In this evaluation, there were major concerns that adequate implementation of P-CIMS would ultimately slow down clinical productivity (inner setting: readiness for implementation). Participants suggested that having a coordinator may circumvent time barriers but perceived that their departments did not have the resources to hire someone to fulfill this role (outer setting: needs and resources for patients with cirrhosis). However, participants (clinical staff) felt that the tool was useful and that they could use it outside their clinic.

The lack of resources to hire a coordinator was because of the lack of buy-in from leadership in their settings for use of clinical tools such as P-CIMS (Inner Setting: Readiness for Implementation). Providers at 2 locations reported that cirrhosis management was not currently a priority for their leadership, and the importance of cirrhosis was secondary to other competing priorities. Health systems have historically established chronic disease priorities based on the overall prevalence of chronic conditions across patients [24]. The apparent lack of leadership support indicates that health informatics tools such as P-CIMS may be more successfully implemented if they are prioritized and championed in the inner clinic setting, or if their importance for patient outcomes is emphasized to leadership.

A second version of the web-based tracking system, based on feedback from this formative evaluation, is under development. In part, this revised version is under development because preliminary results show that the first version of P-CIMS successfully facilitated linkage to liver clinic specialty care for approximately 30% of patients identified as possibly needing care and who were not already being seen in the clinic [13]. The second version will include key upgrades to P-CIMS, including features that facilitate identification and linkage to care of patients living with cirrhosis, as well as improvements in usability. To facilitate these improvements, data will first be pulled from a national EMR instead of a local VHA EMR. This will facilitate the spread of P-CIMS across VHAs nationally. Second, patient identification can be improved to facilitate linkage and retention in care. The new version will identify patients within 30 days of an inpatient diagnosis of cirrhosis, thus increasing the likelihood that liver clinic providers will follow up and retain these patients in care. This version will also identify patients with no formal diagnosis of cirrhosis in the national EMR data, but those with *International Classification of Diseases, Tenth Revision* codes for complications of cirrhosis. These more strategic identification methods are another step in the process of linking patients, who might not otherwise be referred, with specialty care. The final change to patient identification will be to include patients with current or past evidence of positive HCV RNA at any point in VHA care (instead of a recent positive HCV RNA) with fibrosis-4 score >3.25. This will enable providers to monitor hepatocellular carcinoma surveillance of patients who have attained virological cure from direct-acting antiviral treatments. Finally, the user interface will be streamlined to decrease the cognitive load of the end user, streamlining multiple tabs into fewer tabs while providing the necessary information to make clinical decisions. Changes to the second version address several limitations of the initial version. Developers and implementers of similar systems should consider these changes, particularly using a streamlined interface for a busy clinic, for future work in the management of chronic diseases such as cirrhosis.

### Limitations

Although we believe this study is a valuable contribution to the literature examining the implementation of web-based health informatics tools, we do want to address some limitations. First, the study sample size was smaller than that recommended by some standards for formative research evaluations [20]; however, we followed best practices outlined for human factors when evaluating informatics tools [15,25,26]. We also reached thematic saturation related to the identification of the key factors impacting P-CIMS implementation [27]. Within these parameters, our sample size was appropriate, given that the purpose of this study was to identify factors that impacted P-CIMS implementation among a purposive sample of providers of cirrhosis and liver care.

A second limitation was that the provider and care team interviewees in the current sample may have been biased in their perception of P-CIMS as a tool. In the preimplementation phase, sites were chosen based on their perceived ability to implement a cirrhosis management tool. Key characteristics of successful site candidates included established relationships with potential liver clinic champions in the inner setting and external factors that facilitated the implementation of such tools. Furthermore, only 20% (2/10) of the primary care providers were included in the sample. Their perspectives may not have been representative of all VHA primary care providers serving patients with cirrhosis. Barriers and facilitators of implementation may differ in sites without these facilitators. Therefore, these findings may have limited generalizability to both other VA settings and non-VA settings, since data were collected from only 3-VA facilities. These results may also not be generalizable to health care settings that do not have the benefit of a national integrated system of data from EMRs.

Finally, we understand that there is a small likelihood that adverse events may occur with the use of P-CIMS, but our findings from this and the larger evaluation do not indicate that the risk of using the tool is higher than the risks associated with current clinical practices used to track and manage patients with cirrhosis. As reflected in the extant literature, many probable cirrhosis cases are not diagnosed with cirrhosis or seen in specialty liver care. P-CIMS streamlined patient identification and created pathways to improve the coordination and continuity of care [13].

### Impact

P-CIMS is the first informatics tool to leverage EMR data to improve the quality of care for patients with cirrhosis. This tool can be used to inform the design of other clinical informatics tools for a variety of chronic disease conditions that require close tracking and management. However, as is apparent from our qualitative analyses, tools need to be adapted to meet the needs of understaffed clinics or clinics with high workloads. Furthermore, these findings should be used to translate the implementation of health informatics tools for tracking and monitoring chronic diseases in different settings, using tailored implementation strategies as necessary. For instance, future implementation studies could use implementation frameworks such as CFIR, the capability, opportunity, and motivation model, and the behavior change wheel to both: (1) assess major barriers within specific settings, and (2) map these barriers to implementation strategies [28].

### Conclusions

In future studies, patients living with chronic diseases such as cirrhosis should be engaged as key stakeholders during the implementation phase of a novel health informatics tool. It is important to elicit feedback on how health informatics tools can either facilitate or hinder chronic disease management. In this study, lack of resources, lack of support, and competing interests were found to be major barriers to the implementation of this informatics tool. Although these barriers may also be found in other settings, it is important to use implementation frameworks to conduct formative evaluations and adapt strategies that would best translate in those settings. The key element of a formative evaluation in this context is that it enables users’ perceptions of the innovation, in the context of their clinical practices, to be incorporated into the design of a successful, sustainable solution to practice problems.

## Abbreviations

CCTS: Cancer Care Tracking System
CFIR: Consolidated Framework for Implementation Research
EMR: electronic medical record
HCV: hepatitis C virus
P-CIMS: Population-based Cirrhosis Identification and Management System
VA: Department of Veterans Affairs
VHA: Veterans Health Administration
